# Natural liquid betaine dietary supplementation improves growth performance, immuno-antioxidant responses, and stress resistance in Nile tilapia subjected to acute ammonia challenge

**DOI:** 10.1038/s41598-026-47150-0

**Published:** 2026-04-17

**Authors:** Eman N. Elazouny, Asmaa M. El-Nokrashy, Seham El-Kassas, Ahmed M. Abozeid, Basant A. Bakr, Mohamed A. Bauomi, Bahaa Abdella, Radi A. Mohamed

**Affiliations:** 1 Department of Aquaculture, Faculty of Aquatic and Fisheries Sciences, Kafrelsheikh University, Kafr El-Sheikh City, Egypt; 2 Animal, Poultry and Fish Breeding and Production, Department of Animal Wealth Development, Faculty of Veterinary Medicine, Kafrelsheikh University, Kafr El-Sheikh City, Egypt; 3 Institute of Aquaculture, Faculty of Natural Sciences, University of Stirling, Stirling, FK9 4LA UK; 4 Zoology Department, Faculty of Science, Alexandria University, Alexandria City, 21511 Egypt; 5 Fish Production Department, Faculty of Agriculture, Al-Azhar University, Cairo City, Egypt

**Keywords:** Acute ammonia toxicity, Gene expression, Immuno-physiological response, Natural liquid betaine, Nile tilapia, Biochemistry, Physiology, Zoology

## Abstract

This work investigated the effects of natural liquid betaine (NATURA BETAINE) dietary supplementation on physiological performance and alleviation of acute ammonia toxicity in Nile tilapia. Four diets supplemented with 0.0, 0.8, 1.6, and 2.4 mL of liquid betaine /kg diet, representing the experimental treatments B0, B800, B1600, and B2400, respectively, in triplicates were used. Each replicate included 15 Nile tilapia fingerlings (N = 180, initial weight: 9.5 ± 0.429 g). Fish were fed at the rate of 4% of their body weight twice daily for 60 days. At the termination of this feeding trial, the groups B0 randomly divided into two parts B0 − ve and B0 + ve [control negative, and positive, respectively based on ammonia exposure. Then the groups B0 + ve, B800, B1600, and B2400 were subjected to acute ammonia challenge for 72 h, using ammonium chloride at 0.07 g/L. After the 60 days feeding trial, fish received liquid betaine supplemented diets displayed greater growth performance and feed utilization compared to the control group (B0). Additionally, betaine supplementation enhanced serum amylase and lipase concentrations and hematobiochemical metrics. The high dose of liquid betaine (B2400) increased antioxidant enzyme activities and lowered malondialdehyde concentration with increasing serum lysozyme activity and IgM concentrations, and phagocytic activity, and index. At molecular level, betaine dietary supplementation induced upregulations in the mRNA copies of some growth-, immuno- and antioxidant-related genes. Following ammonia exposure, betaine supplementation, particularly the highest dose (B2400) enhanced the cumulative survival rate and mitigated the inflammation signs and tissue degeneration caused by the ammonia challenge in liver, intestine, and gill tissues. In conclusion, dietary supplementation of NATURA BETAINE (at 2.4 mL kg^−1^) enhanced growth performance, optimized physio-biochemical homeostasis, reinforced innate immune and antioxidant responses which may enhance resilience to ammonia stress by improving survival and reducing tissue damage in Nile tilapia, supporting its application as an effective feed additive in aquaculture.

**Supplementary Information:**

The online version contains supplementary material available at 10.1038/s41598-026-47150-0.

## Introduction

Nile tilapia (*Oreochromis niloticus*) is considered a successful fish species for aquaculture due to high production yield, adaptation to environmental changes, and high tolerance against stressors, especially diseases and bad water quality^1^. However, farming intensification increases its exposure to various stressors which may impact its health, welfare, and performance^2^. These challenges include changes in water parameters, especially temperature, salinity, and the total ammonia nitrogen^3–5^.

Ammonia, a dissolved gas in water, is highly toxic in its forms (unionized (NH_3_) or ionized ammonia (NH_4_^+^))^6^. Both acute and chronic exposure to ammonia can negatively influence fish health and physiological status due to tissue damage that results from ammonia-associated oxidative stress^7^. Besides, Acute exposure to ammonia reduced total antioxidant capacity^8^, increased circulating glucose and cortisol and altered plasma contents of aspartate aminotransferase (AST) and aminotransferase (ALT)^9^. In addition, at molecular level, exposure to ammonia has been shown to alter expression levels of inflammation-regulatory genes inducing an immunosuppression effect that was expressed in lowering the plasma concentration of complement proteins, lysozyme, and immunoglobulins^8,10,11^. In Nile tilapia, exposure to ammonia has been demonstrated to damage gill tissue and disrupt its function. It resulting in impaired respiration and oxygen transportation which in turn altered the acid–base balance and causing harm to hepatic, kidney, and intestinal tissues^12,13^. Moreover, prolonged exposure to ammonia depressed Nile tilapia growth performance, reduced the efficiency of feed utilization, and impaired immunity, findings that were attributed to increased generation of reactive oxygen species (ROS)^14,15^. These consequences of exposure to ammonia may compromise Nile Tilapia’s health, welfare, and growth, making ammonia toxicity a serious challenge for sustainable tilapia production and raising the need to explore various approaches to tackle this problem. Therefore, dietary supplementation with a balanced, high-quality diet containing essential macro- and micronutrients and feed additives becomes a priority to ensure higher production^16^. Such approaches may enhance basal preparedness which would contribute to lowering incidence of diseases^17^ and improving the overall welfare of tilapia. Consequently, feed additives such as probiotics, prebiotics, herbal extracts, plant derivatives, attractants, and enzymes are used during aquafeed manufacturing to boost feed efficiency, digestion, and absorption and to ultimately improve growth performance and enhance immune response under different farming conditions^18–20^.

Betaine, which is a powerful donor of methyl group (CH_3_) can replace the other methyl group donors such as choline and methionine, which can assist fish maintaining internal physiological condition and in improving growth performance^21^. Betaine’s effects include stabilization of the cellular acid–base balance and contribution to the methylation reactions crucial for the amino acid and energy metabolism^22^. These beneficial effects of betaine may augment its potentiate role in overcoming negative influences of ammonia toxicity on survival rate and tissue degeneration in Nile tilapia. There are two types of betaines: natural and synthetic^23,24^. Synthetic betaine (betaine HCL) is a trimethyl product of glycine amino acid and is obtained from choline^25^. Whereas the natural betaine is primarily derived from the processing of sugar beets^23^ that contain the highest amounts of betaine^26^. Many differences exist between natural and synthetic betaine in the form of extraction, processing, composition, application dose, toxicity^27^, and gut absorption^23^. In aquaculture, previous studies on betaine showed a wide range of applications in the aquafeed sector^21^ including physiological and metabolic functions^28^ in fat metabolism, and enhancing immune response in both fish and shrimp^29^. Besides, dietary incorporation of betaine resulted in elevated feed consumption and growth yield of various aquatic organisms^27^. For Nile tilapia, betaine supplementation increased feed intake^30^, lipid deposition, final production, feed conversion, weight growth, and feed digestibility^25^;^31^. An extensive amount of research work was carried out on synthetic betaine (betaine HCL, solid form), whereas very little is known about the effects of natural betaine (soluble form). Besides, studying the impacts of natural betaine supplementation on growth performance, efficiency of feed utilization, immune and antioxidant parameters as well as the ammonia-associated tissue degeneration could provide an integrated understanding of the potential role of natural betaine in reducing the impact of ammonia toxicity in Nile tilapia. Therefore, the present study aimed to investigate the effect of soluble natural betaine (NATURA BETAINE) on growth metrics, feed utilization and absorption, physio-biochemical profile and immuno-antioxidant responses of Nile tilapia (*Oreochromis niloticus*) under normal farming as well as the tissue degeneration-associated with acute ammonia challenge.

## Materials and methods

### Ethical approval

The experimental procedures of the current study adhered to the Egyptian legislation on ethics in fish use and handling and were approved by the Committee of Aquatic Animal Care and Use in Research at the Faculty of Aquatic and Fisheries Sciences, Kafrelsheikh University, Egypt (approval number: IAACUC-KSU-017-2020). The study is reported in accordance with ARRIVE guidelines for animal research and all experiments were performed in accordance with relevant guidelines and regulations.

### Diet preparation, experimental design, and fish husbandry

A private tilapia farm (Baltem district, Kafr El-Sheikh governorate, Egypt) provided the mono-sex Nile tilapia (*Oreochromis niloticus*) fingerlings (2.5 month) utilized in this experiment. Fish were maintained in glass aquariums for acclimatization to the laboratory conditions for 14 days with water quality parameters maintained at 26.48 ± 1.07 °C for temperature, 7.8 ± 0.31 for pH, 7.55 ± 0.74 mg/L for dissolved oxygen, and 0.045 ± 0.006 mg/L for total ammonia nitrogen. During acclimatization, Nile tilapia fingerlings received a commercial diet containing 30% crude protein and were fed at a rate of 4% of the total body mass, twice per day (09:00 am and 4:00 pm). After acclimatization, 180 apparently healthy Nile tilapia fingerlings, with an average body weight of 9.5 ± 0.429 g, were selected and allocated at random in triplicates at a density of 15 fingerlings for each 90-L aquarium (80 × 40 × 45 cm). The fish were randomly categorized into 12 glass aquaria to form four test groups in the following manner:

I. The first (control) group (B0) was given only a commercial basal diet (Table 1) manufactured by a local aquafeed factory, Kafr El-Sheikh, Egypt.
II. The second group (B800) of fish received a basal diet supplemented with liquid betaine at 0.8 mL/Kg diet [NATURA BETAINE (91–99% betaine on dry matter basis)—Natura Feed Ingredients, Atlanta, trademarks of CSP Inc., USA] as per the instructions of the manufacturer and to evaluate commercially relevant inclusion levels (Supplementary file 1).
III. The third group (B1600) of fish was fed a basal diet enriched with liquid betaine at 1.6 mL/Kg diet according to the guidelines of the supplier.
IV. The fourth group (B2400) of fish was fed a basal diet enriched with liquid betaine at 2.4 mL/Kg diet following the supplier’s recommendation.

**Table 1:** Ingredients and chemical analysis of basal experimental diet.

Ingredient	Kg	Proximate composition^b^	(%)
Fishmeal	6.000	DM %	89.10
Soybean meal	37.40	Moisture %	10.90
Corn meal	27.00	Crude protein %	30.02
Corn gluten	6.100	Fat %	3.580
Wheat bran	7.000	Fiber %	4.880
Rice bran	11.80	Ash %	6.760
Fish oil	1.700	Nitrogen free extract (NFE)	43.5
Vitamins and minerals mix^a^ (g/kg)	3.00	Digestible energy (DE)	2900 kcal/kg
Total	100		

^a^Each 1 kg contains Vit. A 4.8 I.U.; Vit. D2 0.8 I. U; Vit E 4.0 g; Vit. K 0.8 g; Vit B 0.49; Vit. B2 1.6 g; Vit. B6 0.6 g; Vit. B12 4 mg; Pantothenic acid 49 g; Nicotinic acid 8 g; Folic acid 400 mg; Biotin 20 mg; Copper 4.0 g; Iodine 0.4 g; Iron 12 mg; Manganese 22 g; Zinc 22 g and Selenium 0.04 g.

^b^The chemical analysis of feed was received from the commercial source.

For each treatment, the supplemented volume of liquid betaine (0.8, 1.6, and 2.4 mL for the B800, B1600, and B2400 treatments, respectively) was integrated into each 1 kg of experimental diet. Following feed manufacturing, the liquid betaine was mixed with distilled water to a total volume of 10 mL and then sprayed onto the feed. The control group (B0) was sprayed with 10 mL of distilled water only. Subsequently, all diets were dried at ambient temperature for 24 h, after which they were stored at -20˚C until required. The basal diet was formulated to meet the nutrient requirement of tilapia according to National Research Council requirements of fish and shrimp^32^.

Aeration was provided for each aquarium via air stones. To get rid of the fish waste, a mechanical filter was applied and cleaned daily. Approximately 30% of the water was changed every 2 days using overnight-kept water to restore the water volume to 90 L. Throughout the experiment, the photoperiod was adjusted to a 12 h light/12 h dark cycle. Experimental diets were provided to fish at a rate of 4% of body weight two times per day, at 08:00 and 14:00 h, for 60 days. The uneaten feed was gathered, then dried, and weighed after each feeding to calculate the net feed intake. Mortality data were documented daily; the mortality rate was 0% during the entire experiment prior to ammonia exposure.

To evaluate the water quality, twice per week in each tank, pH levels were assessed by using a HACH PHC725 pH meter. While dissolved oxygen (DO) and temperature were recorded with an OxyGuard Handy Polaris meter which immersed at 10 cm below the water surface to ensure accurate readings^33^. Also, from each aquarium’s midpoint, water samples (20 mL) were taken to measure the total ammonia nitrogen (TAN) via a portable photometer (Martini MI 405) according to the methods reported by Eaton et al.^34^ and Rice et al.^35^.

### Fish growth and biometric indices

The initial weights (g) of Nile tilapia fingerlings were reported at the beginning of the experiment. Then, the weight of fish was recorded at two-week intervals to balance temporal resolution with animal welfare and to adjust the feeding rate by calculating the amount of feed as a percentage of tank biomass. At the end of the 60-day experimental duration, Nile tilapias, from each replicate, were fasted for 6 h then were harvested by using appropriate nets and anesthetized using eugenol (clove oil, Merck, Germany, ≥ 99% purity) at 50 mg L^−1^ as recommended in the manufacture instructions. Clove oil was prepared as a stock solution (1:10 in 95% ethanol) and was diluted in water (27 °C) in open tank to make an anesthetic bath. After harvest, each individual fish was transferred to the clove oil-anesthetic bath and was monitored for about ~ 1.5 min until the loss of equilibrium and the stop of movement was achieved. To assess growth performance, each fish was individually weighed using a digital balance to calculate the final weight. Prior to weighing, the fish from each aquarium were blotted using clean and sterile filter papers to remove excess water. To determine each fish’s total length (L), a measuring board was utilized starting from the most anterior tip of the fish’s mouth toward the extended tip of the caudal fin. The assessment of growth performance, feed utilization, and biometric indices was carried out using the following calculation methods:

$$Specific\;growth\;rate (SGR \% /day) = 100\times (lnW1-lnW0) / the\;experimental\;period\;(days)$$

$$Body\;weight\;gain \left( {BWG} \right) = final\;body\;weight \left( {W1} \right)/g{-}initial\;body\;weight \left( {W0} \right)/g$$

$$Weight\;gain\;rate\;(WG \%) = (W1 - W0)/W0 \times 100$$

$$Protein\;efficiency\;ratio\;(PER) = weight\;gain\;(g) / protein\;intake\;(g).$$

$$Condition\;factor\;(K) = 100 \times (W1/L3)$$

$$Feed\;conversion\;ratio\;(FCR) = feed\;intake\;(g) / BWG\;(g)$$

$$Viscero-somatic\;index\;(VSI) = 100 \times (intestine\;weight / W1)$$

$$Hepato-somatic\;index\;(HIS) = 100 \times (liver\;weight / W1)$$

$$\begin{aligned} & Survival\;rate\left( \% \right) = \\ & \quad 100 \times \left( {the\;number\;of\;fish\;at\;the\;end\;of\;the\;trial/the\;number\;of\;fish\;at\;the\;start\;of\;the\;trial} \right). \\ \end{aligned}$$

where is W0 = initial body weight, W1 = final body weight, WG = weight gain, lnW1 = natural log of final body weight, lnW0 = natural log of initial body weight, and L^3^ = cubic fish length. To calculate VSI and HIS, the fish were dissected and the liver and intestine weight was recorded for each fish.

### Blood and tissues samples and serum separation

From the caudal vein of each fish (9 fish/treatment “3 per tank”), blood samples were collected in vacuum tubes at the termination of the current feeding trial (before ammonia exposure). The obtained blood was collected into two sterile tubes: the first tube contained ethylenediaminetetraacetic acid (EDTA) as an anticoagulant and was used for hematological assays. The other tube did not include anticoagulants and was used for blood serum collection by centrifuging the clotted blood in an Eppendorf centrifuge (SIGMA 4–16 K refrigerated centrifuge, Sigma Laborzentrifugen, Germany) at 3000 rpm at 4 °C for a period of 15 min. Then the supernatant serum was carefully removed, placed into plastic Eppendorf tubes, and stored at − 20 °C for further analysis of serum biochemical parameters, digestive enzymes activities, antioxidants, and immune response parameters. After blood sampling, fish samples (6 fish/treatment) were dissected and specimens from liver were collected from each fish, snap-frozen in liquid nitrogen, and kept at − 80 °C for RNA extraction and qRT-PCR analysis.

### Hematological analysis

Total leukocyte (WBCs) and erythrocyte (RBCs) counts, hemoglobin content (Hb) by means of the cyanomet hemoglobin method and Drabkin’s solution, while the packed cell volume (PCV) using the microhematocrit technique, were measured in line with the technique previously described by^36^. Also, thin blood smears were made, allowed to air-dry, fixed in methanol for a duration of 3–5 min, stained with Gimsa for 8–10 min, and after that set aside to dry completely to determine differential leukocytic count (lymphocyte, heterophil and monocyte). The WBCs (one hundred cells for each blood smear) were counted according to^36^.

### Analysis of serum biochemical profile, digestive enzymes activity, antioxidant capacity, and lysozyme activity

As described by Reitman and Frankel^37^, alanine aminotransferase (ALT) and aspartate aminotransferase (AST) activities were evaluated colorimetrically at a wavelength 540 nm using commercially available kits according to manufacture instructions. As demonstrated by Doumas and Biggs^38^ and Doumas et al.^39^, total protein and albumin were determined, respectively. Globulin content was calculated mathematically by subtracting albumin from total protein. Total cholesterol and triglyceride were analyzed in serum as per the company’s guidelines of commercial available kits (CHOD-PAP and GPO-PAP), respectively^40^. Serum urea and creatinine were determined by the colorimetric method using available commercial kits^41^. According to Trinder^42^ serum glucose concentration was determined using accessible commercial enzymatic PAP kits from Bio-Merieux (France).

Serum activities of amylase and lipase digestive enzymes (9 fish/treatment) were analyzed using the diagnostic reagent kits (Cusabio Biotech Co. Ltd., Wuhan, Hubei, China) according to the company’s instructions. Lipase and amylase activities were measured using colorimetric analysis at wavelengths of 580 nm and 660 nm, respectively^43,44^.

Using ELISA kits (Inova Biotechnology, China) and the microplate ELISA reader at the wavelength 450 nm, the activities of superoxide dismutase (SOD), and catalase (CAT), and the concentration of malondialdehyde (MDA) were measured in 9 randomly selected fish for each treatment adopting the method explained in^43^.

To study the impact of dietary betaine on fish immune status, 9 fish were randomly chosen from each treatment group (9 fish/treatment). Following the procedure stated by^45^, the serum lysozyme activity was assayed by microplate ELISA reader at 450 nm wavelength. Determination of immunoglobulin M (IgM) was done employing ELISA via a commercial kit from Cusabio (Wuhan, Hubei, China) following the guidelines drawn by the manufacturer^46^. In accordance with the method explained by^47^, leucocyte phagocytic activity against *Candida albicans* was assessed in vitro. Phagocytic activity was determined by calculating the percentage of phagocytes engulfing yeast relative to the total number of phagocytes. While the phagocytic index was determined by dividing the number of phagocytized yeast cells by the number of phagocytes already engulfed the yeast cells.

### RNA extraction, cDNA synthesis, and quantitative real-time PCR (qRT-PCR)

The anesthetized tilapia was subjected to spinal cord crushing euthanasia to ensure fish death. Following dissecting the fish and isolating liver tissues, samples (6 fish/treatment) were collected in 2 mL sterile Eppendorf tubes. The tissues were flash-frozen in liquid nitrogen and kept at − 80 °C pending further laboratory testing. Total RNA was extracted from 50 mg of liver tissue applying Trizol (iNtRON Biotechnology, Inc., Korea) as per the company’s instructions. The quantity and quality of the extracted RNA were verified using a Nanodrop (UV–Vis spectrophotometer Q5000/Quawell, USA). Subsequently, a SensiFAST cDNA synthesis kit (Bioline, United Kingdom) was used to make complementary DNA (cDNA) in accordance with the manufacturer’s guidelines.

To amplify the chosen genes in tilapia (Table 2)*; growth hormone (gh), insulin growth factor I (igf-I), superoxide dismutase (sod), catalase (cat),* and *interleukins 6 and 8 (il-6 & il-8) genes* plus the β-actin as a housekeeping gene (an internal reference gene), targeted primers were employed. Using the quantitative real-time PCR system (Stratagene MX300P), gene expression was analyzed. SYBR green master mix TOP real preMIX SYBR Green qPCR master mix (Enzynomics, cat. RT 500) was used to quantify the selected genes. The thermocycling protocol began with 95 °C for 30 s, followed by 40 cycles of denaturation for 60 s at 63 °C, and finally annealing for 60 s at 60 °C. The expression of each gene was normalized to the β-actin within each sample. After proving PCR efficiency at approximately 100%, data were then determined using the 2^^−∆∆CT^ technique^48^.

**Table 2:** Primers used for qRT-PCR analysis.

Gene	Primer sequence 5′-3′ (F = forward- R = reverse)	Annealing temp. (°C)	Gene Bank accession No	slope	Efficiency%	Reference
*gh*	F: GTTGTGTGTTTGGGCGTCTC R: CAGGTGCGTGACTCTGTTGA	60	HM565014.1	− 3.384	97.473	Abo-Raya et al.^19^
*igf-I*	F: TTGTCTGTGGAGAGCGAGGCTT R: CAGCTTTGGAAGCAGCACTCGT	62	XM_003448059	− 3.400	96.842	Yuan et al.^49^
*cat*	F: TTTGCACGTTTACAGCCGTC R: CTGATGGCTCGAATAATGGTCC	60	JF801726.1	− 3.398	96.920	Yuan et al.^49^
*sod*	F: ACGTGACAACACAGGTTGCT R: AGTCCCGTTTGATTGCCTCC	58	JF801727.1	− 3.471	94.134	Yuan et al.^49^
*il-6*	F: AAACCAATTCCTTCTGGCCCT R: TCACCTGAGAAGTCACCTGC	56	XM_019350387.2	− 3.342	99.169	Wang et al. (2024)^90^
*il-8*	F: CTGTGAAGGCATGGGTGTG R: ATCACTTTCTTCACCCAGGG	58	NM_001279704	− 3.345	99.046	Yuan et al.^49^
*β-actin*	F: GTGCCCATCTACGAGGGTTA R: CTCCTTAATGTCACGCACGA	60	XM_003443127	− 3.394	97.078	Qiang et al. (2014)^89^

*Growth hormone (gh), insulin growth factor 1 (igf-1), superoxide dismutase (sod), catalase (cat), Interleukin-6 (il-6), interleukin-8 (il-8), and housekeeping (β-actin) gene.*

### Acute ammonia exposure

After the termination of the 60-day betaine-feeding period and blood and tissue sampling, the control experimental group (B0) was equally divided into B0 − ve (control negative) and B0 + ve (control positive); B0-ve denotes absence of both acute ammonia exposure and natural liquid betaine supplementation while B0 + ve refers to occurrence of (positive) ammonia exposure without natural liquid betaine supplementation. Each treatment contains 20 fish (2 replicates, 10 fish / replicate). The groups (B0 + ve, B800, B1600, and B2400) were exposed to an acute ammonia challenge for 72 h, using ammonium chloride (99.998% trace metals basis, Sigma, USA) which was added to water at 0.07 g/L every 7 h for a continuous 72 h^4^. Water pH was maintained at a range from 7.89 to 8.1 during the period of ammonia exposure. An ammonia ion-specific meter (Martini MI 405) was used to measure the concentration of ammonia at 1- and 4-h post-addition and was stabilized following the daily water exchange. Total ammonia nitrogen level was maintained around 5 mg/L during the 72-h challenge period^50^ and the level was maintained by adding extra ammonium chloride solution. B0 − ve group was kept as a control and was reared under normal experimental condition. After the end of the 72-h challenge, the fish were incessantly observed for additional 10 days, and the daily fish mortality was determined, and the cumulative survival was estimated.

### Histomorphology examination following acute ammonia challenge

After acute ammonia challenge, anesthetized tilapia was subjected to spinal cord crushing euthanasia to ensure fish death. The fish’s liver, gut, and gills were dissected (6 fish/treatment) and fixed in 10% neutral buffered formalin over a span of 12–24 h. The dissected organs were subsequently processed and wrapped in paraffin wax after being put in tissue cassettes. At 5 μm, an implanted sample was cut with a microtome. Hematoxylin and eosin (H&E) was used to stain the slices^51^. A light microscope equipped with a camera (Lecia DM750 P) was used to view and capture images for the stained slides. Moreover, the effect of the acute ammonia challenge following the supplementation of natural liquid betaine on intestinal histomorphometry was explored by measuring height of villus (from tip to base), tip width, width at the crypt/villus interface, and muscularis layer thickness. The quantitative evaluations were performed manually on photomicrographs software via ImageJ with two distinct observers^52^.

### Statistical analysis

All collected data were assessed for normality of distribution, which was then verified by analysis of the residuals, using the Shapiro–Wilk and Levene’s tests. Percentage data went through an arcsine transformation prior to analysis. Statistical evaluation of the data was conducted using GraphPad Prism 6 (GraphPad Prism v6.0, San Diego, CA, USA). Data were analyzed using a one-way ANOVA followed by Tukey’s multiple comparison tests employed to identify significant differences between the means. Polynomial regression analysis was applied to discover the cubic and quadratic influences of different concentrations of supplemented betaine on weight gain, final body weight, and feed conversion ratio. Log-rank (Mantel Cox) was employed for statistical calculation of cumulative survival of Nile tilapia fingerlings challenged with ammonia toxicity. Comparisons were considered significant at *P* < 0.05.

## Results

### Fish growth performance, feed utilization efficiency, biometric indices, and digestive enzymes activity

**Fig. 1:**
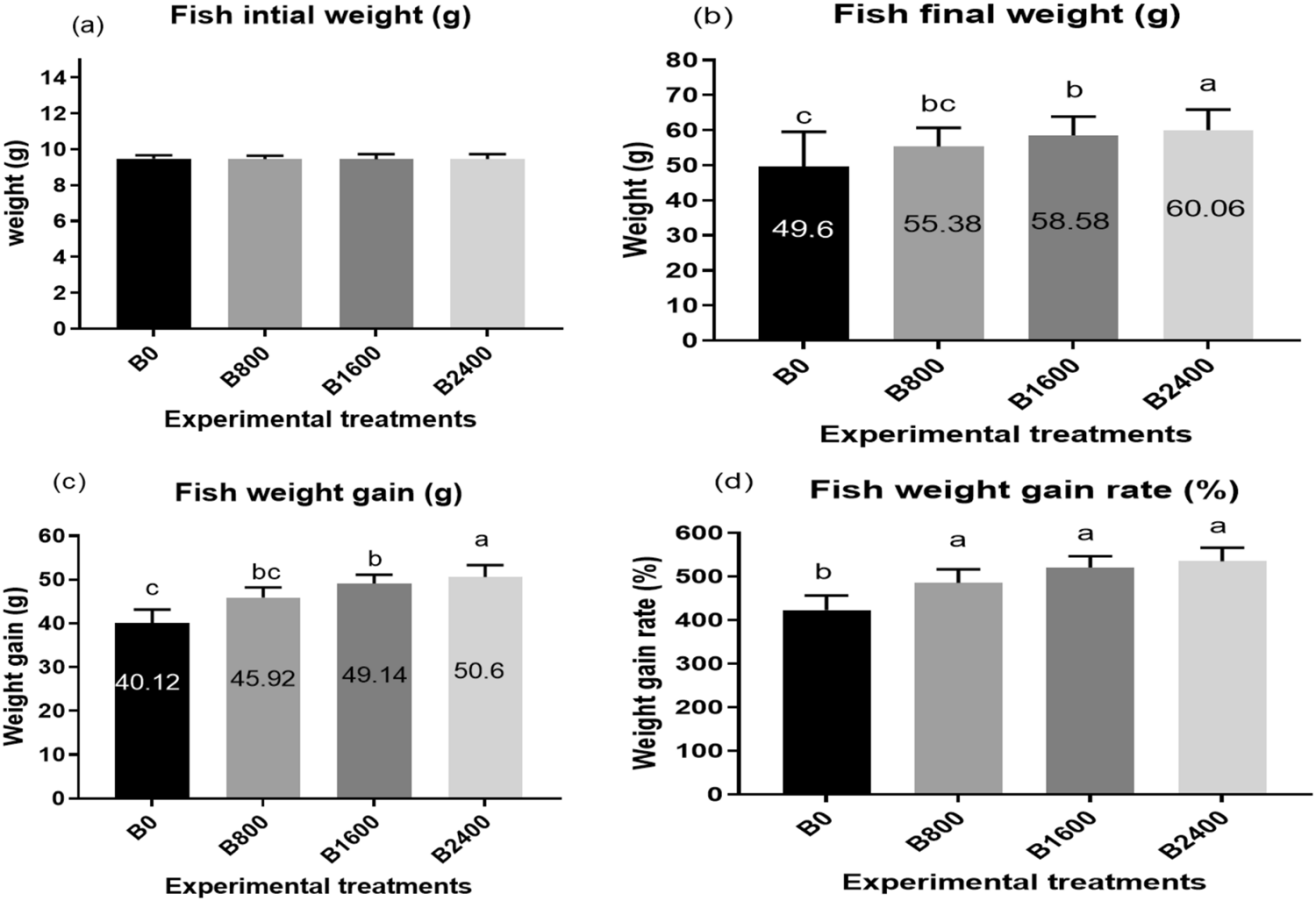
Growth performance (**a**–**d**) of fish fed experimental diets. The results were expressed as mean ± SE and the different letters indicate statistical significance at *P* < 0.05.

**Fig. 2:**
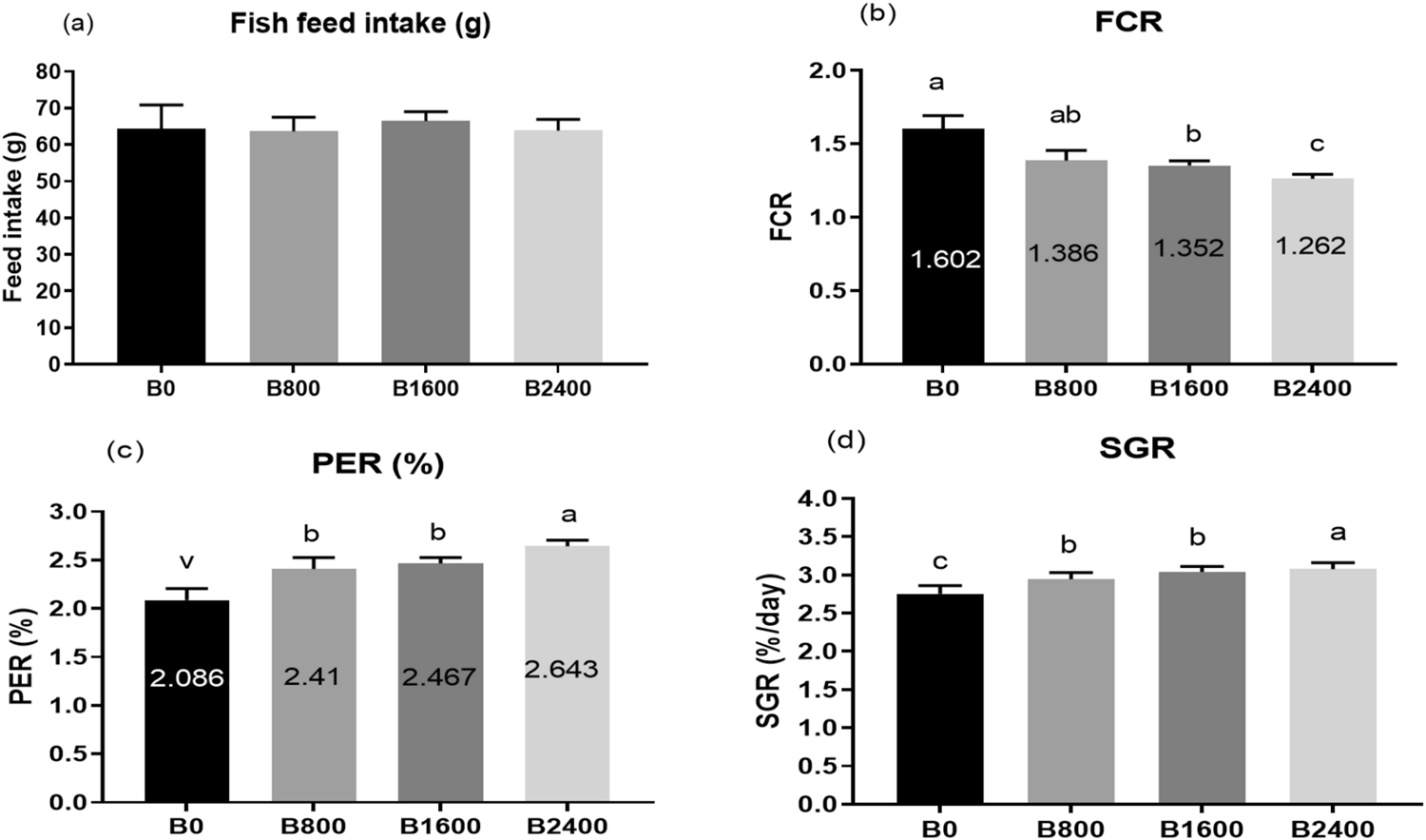
Feed utilization efficiency (**a**–**d**) of fish fed experimental diets. The results were expressed as mean ± SE and the different letters indicate statistical significance at *P* < 0.05. SGR = specific growth rate; FCR = feed conversion ratio; PER = Protein efficiency ratio.

**Fig. 3:**
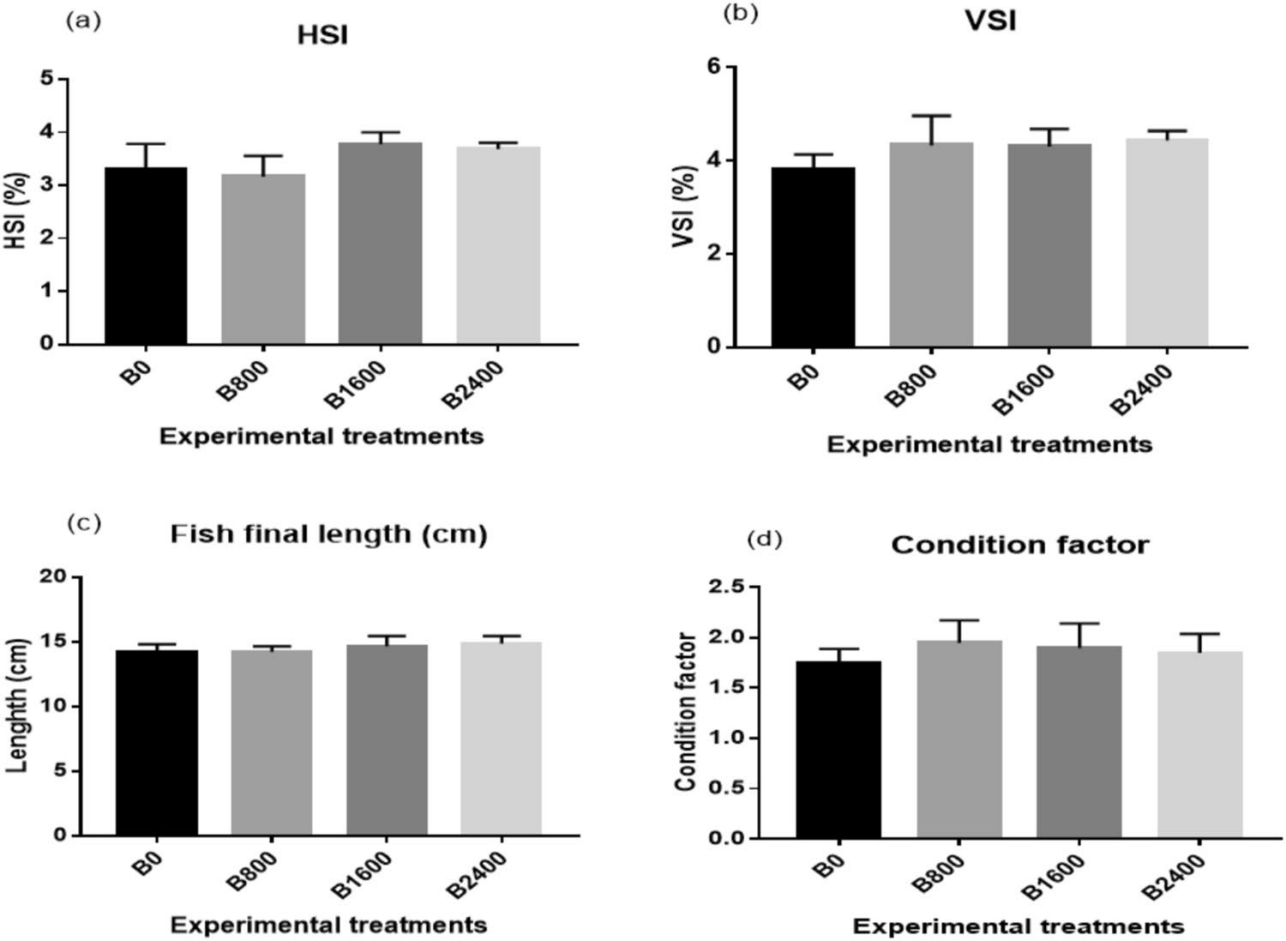
Biometric indices (**a**,**b**), fish body length (**c**) and condition factor (**d**) of Nile tilapia fingerlings fed experimental diets. The results were expressed as mean ± SE and the different letters indicate statistical significance at *P* < 0.05. HSI = hepatosomatic index; VSI = viscerosomatic index.

**Fig. 4:**
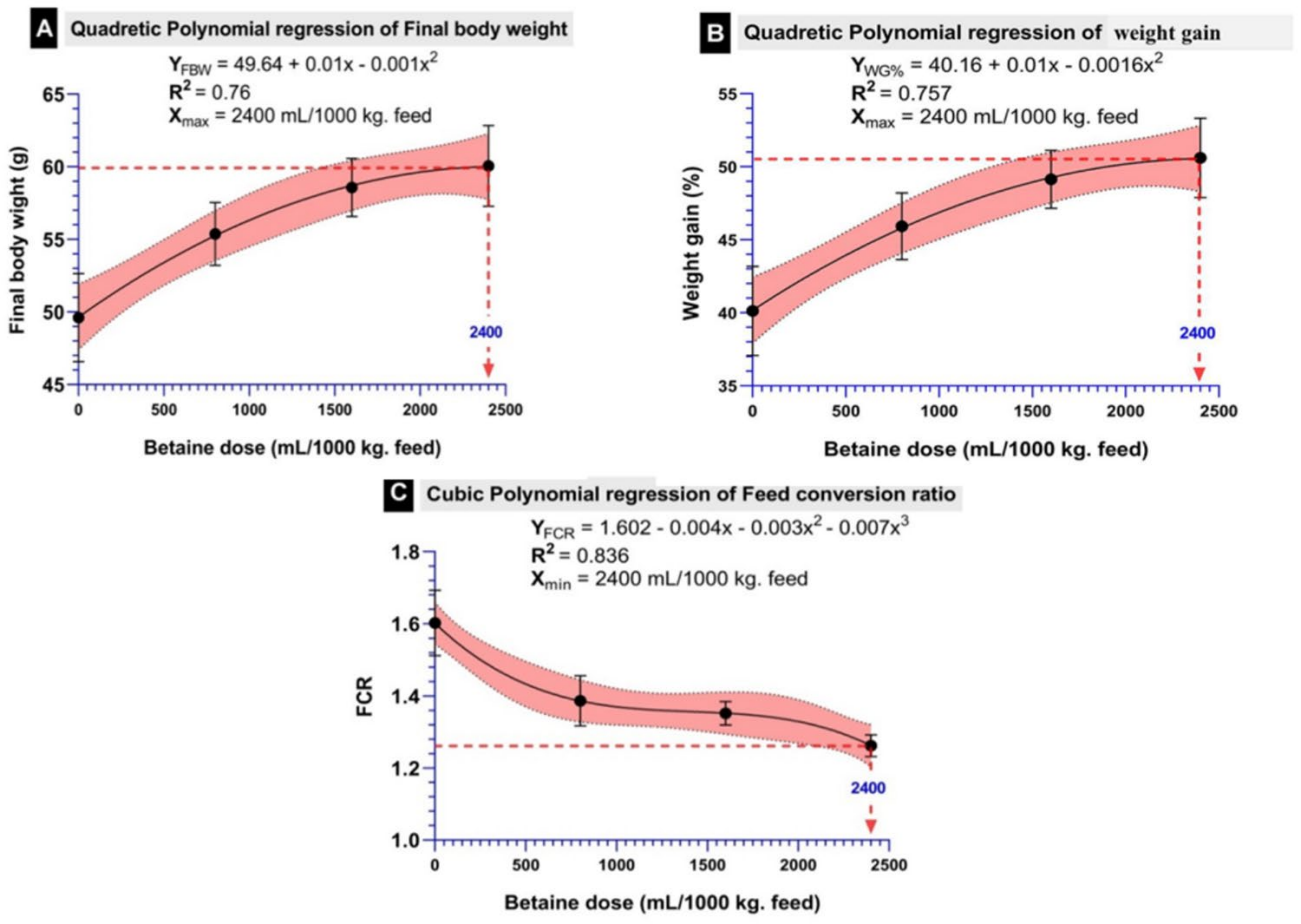
Significant quadratic and cubic relationships and polynomial regressions analysis (*P* < 0.05) between final body weight (**A**), weight gain (**B**) and feed conversion ratio of Nile tilapia and dietary levels of liquid betaine.

At the termination of the trial, there was a substantial increase (*P* < 0.001) in the weight gain and final body weight with the rise in the natural betaine dose, reaching the maximum values of 60.1 g and 50.6 g, respectively, in the B2400 group (Figs. 1b, c and 4a, b). Similarly, betaine supplementation resulted in a significant upsurge (*P* < 0.001) in the weight gain rate compared to the control (B0) (Fig. 1d). There were no substantial modifications in the values of initial weight and feed intake between dietary groups (*P* > 0.05) (Figs. 1a and 2a, 3).

In terms of FCR, there was a substantial reduction in its values in fish fed the betaine diet with respect to the control treatment (*P* < 0.001), with the lowest value (around 1.25) displayed in the B2400 group (Figs. 2b and 4c). Besides, when supplementing the basal diet with liquid betaine, there was a significant enhancement in the levels of PER and SGR (Fig. 2c, d). Regarding the biometric indices, dietary liquid betaine supplementation did not alter the HSI, VSI, FL, and CF (*P* > 0.05) (Fig. 3), and the survival rate was 100% in all treatment groups. Significant quadratic and cubic relationships and polynomial regression analysis (Fig. 4) between final body weight (Fig. 4A), weight gain (Fig. 4B) and feed conversion ratio (Fig. 4C) revealed that the best dietary level of liquid betaine for Nile tilapia is 2.4 ml/kg diet.

**Fig. 5:**
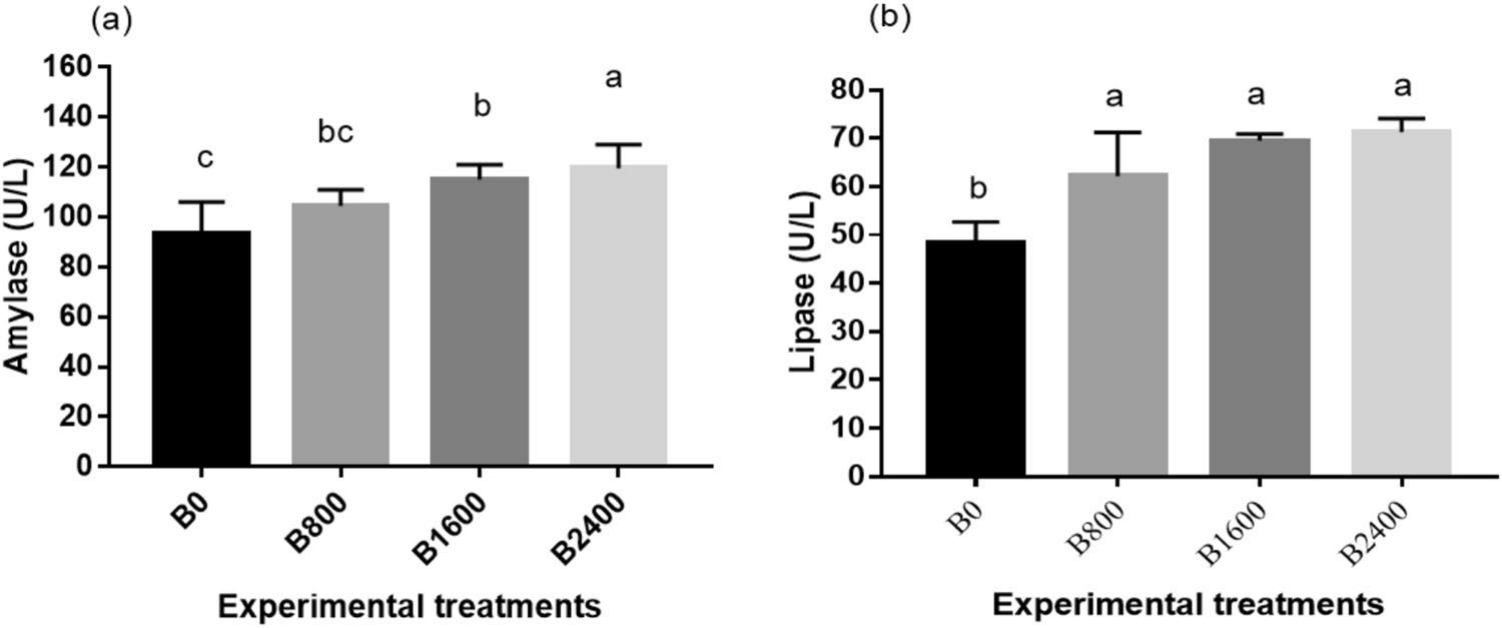
Digestive enzyme activity (**a** amylase, **b** lipase) of fish fed experimental diets**.** The results were expressed as mean ± SE and the different letters indicate statistical significance at *P* < 0.05.

Figure 5 shows the results of digestive enzymes activity in the serum of fish. The amylase activity was considerably (*P* = 0.031) elevated in the betaine treated groups as opposed to the control with the highest level displayed in the B2400 group (Fig. 5a). Similarly, the treatment groups (B800, B1600, and B2400) demonstrated substantial improvement (*P* = 0.0026) in the activity of lipase in contrast to B0 (Fig. 5b).

### Hematological and biochemical parameters

The impacts of dietary liquid betaine supplementation on the hematological standards of Nile tilapia are summarized in Table 3. The values of RBC and WBC count were significantly augmented (*P* < 0.05) with the increase of dietary betaine levels up to 2.4 ml kg^−1^ diet, whereas the Hb, PCV, lymphocyte, heterophil, and monocyte values were not markedly (*P* > 0.05) influenced by betaine inclusion (Table 3).

**Table 3:** Hematological parameters (mean ± SE) of fish fed experimental diets.

	B0	B800	B1600	B2400	*P*-value
RBCs (× 10^6^/mm^3^)	1.245 ± 0.026^b^	1.340 ± 0.023^ab^	1.330 ± 0.012^ab^	1.400 ± 0.046^a^	0.036
Hb (g/dL)	8.950 ± 0.087	9.350 ± 0.144	9.450 ± 0.375	10.10 ± 0.346	0.087
PCV (%)	29.30 ± 1.617	26.35 ± 1.862	28.30 ± 1.401	25.65 ± 1.490	0.368
WBCs (× 10^3^/mm^3^)	24.25 ± 0.549^b^	25.70 ± 1.732^ab^	27.60 ± 0.808^ab^	29.60 ± 0.982^a^	0.042
Lymphocyte (%)	93.33 ± 1.764	91.33 ± 0.667	92.67 ± 2.667	94.67 ± 2.028	0.680
Heterophil (%)	3.000 ± 0.208	2.967 ± 0.145	3.133 ± 0.120	3.233 ± 0.203	0.689
Monocyte (%)	2.5 ± 0.281	2.0 ± 0.011	1.5 ± 0.282	1.5 ± 0.274	0.063

Means within the same row lack common superscripts are significantly different at *P* < 0.05. Red blood cells (RBCs), Hemoglobin (Hb), Packed cell volume (PCV), White blood cells (WBCs).

The serum levels of TP and globulins in fish fed betaine-based diets significantly improved (*P* = 0.018), compared to the control (Table 4). Conversely, the values of ALT, triglyceride, and cholesterol were significantly declined (*P* < 0.05) with rising dietary betaine concentrations, with the lowest values displayed in the B2400 group. However, the AST, albumin, creatinine, urea, and glucose levels were not notably altered by dietary supplementation (*P* > 0.05) (Table 4).

**Table 4:** Biochemical parameters (mean ± SE) of fish fed experimental diets.

	B0	B800	B1600	B2400	*P*-value
ALT (U/l)	52.0 ± 1.155^a^	48.5 ± 2.598^ab^	40.5 ± 3.753^ab^	38.0 ± 2.309^b^	0.017
AST (U/l)	195 ± 31.75	182 ± 33.84	125 ± 16.74	104 ± 12.70	0.097
TP (g/dL)	3.967 ± 0.167^b^	4.233 ± 0.203^ab^	4.433 ± 0.202^ab^	4.967 ± 0.088^a^	0.018
Albumin (g/dL)	2.033 ± 0.291	2.067 ± 0.145	2.000 ± 0.101	2.167 ± 0.033	0.909
Globulins (g/dL)	1.933 ± 0.177^b^	2.133 ± 0.145^ab^	2.433 ± 0.176^ab^	2.800 ± 0.116^a^	0.020
Triglyceride (mg/dL)	206.3 ± 3.002^a^	193.2 ± 2.858^ab^	186.7 ± 5.398^b^	168.1 ± 5.167^b^	0.002
Cholesterol (g/dL)	156.4 ± 4.677^a^	137.3 ± 3.839^ab^	131.8 ± 5.340^b^	127.3 ± 4.041^b^	0.008
Creatinine (mg/dL)	0.379 ± 0.035	0.400 ± 0.006	0.376 ± 0.048	0.332 ± 0.006	0.475
Urea (mg/dL)	16.95 ± 1.010	18.35 ± 0.664	15.45 ± 1.241	15.10 ± 1.039	0.168
Glucose (mg/dL)	54.37 ± 2.384	56.70 ± 4.869	57.00 ± 5.632	61.63 ± 4.174	0.711

Means within the same row lack common superscripts are significantly different at *P* < 0.05. Alanine aminotransferase (ALT), Aspartate aminotransferase (AST), Total protein (TP).

### Water quality analysis

Table 5 displays the physiochemical parameters of water. The assessed parameters of water quality did not significantly differ among groups; they fell between 6.17–6.27 mg/L, 7.87–8.14, 26.03–26.1 °C, and 0.28–0.29 mg/L for DO, pH, temperature, and TAN, respectively.

**Table 5:** Water quality parameters (mean ± SE) of fish fed experimental diets.

	B0	B800	B1600	B2400	*P*-value
DO (mg/L)	6.193 ± 1.187	6.267 ± 1.120	6.233 ± 1.125	6.167 ± 1.176	0.947
Temperature (°C)	26.03 ± 1.233	26.1 ± 1.057	26.1 ± 1.257	26.07 ± 1.333	0.719
pH	7.867 ± 0.376	7.967 ± 0.286	8.101 ± 0.422	8.143 ± 0.220	0.915
TAN (mg/L)	0.284 ± 0.034	0.276 ± 0.038	0.290 ± 0.020	0.288 ± 0.011	0.982

Means within the same row with different superscripts are significantly different (*P* < 0.05). Dissolved oxygen (DO), Total ammonia nitrogen (TAN).

### Antioxidant capacity and Immune response

**Fig. 6:**
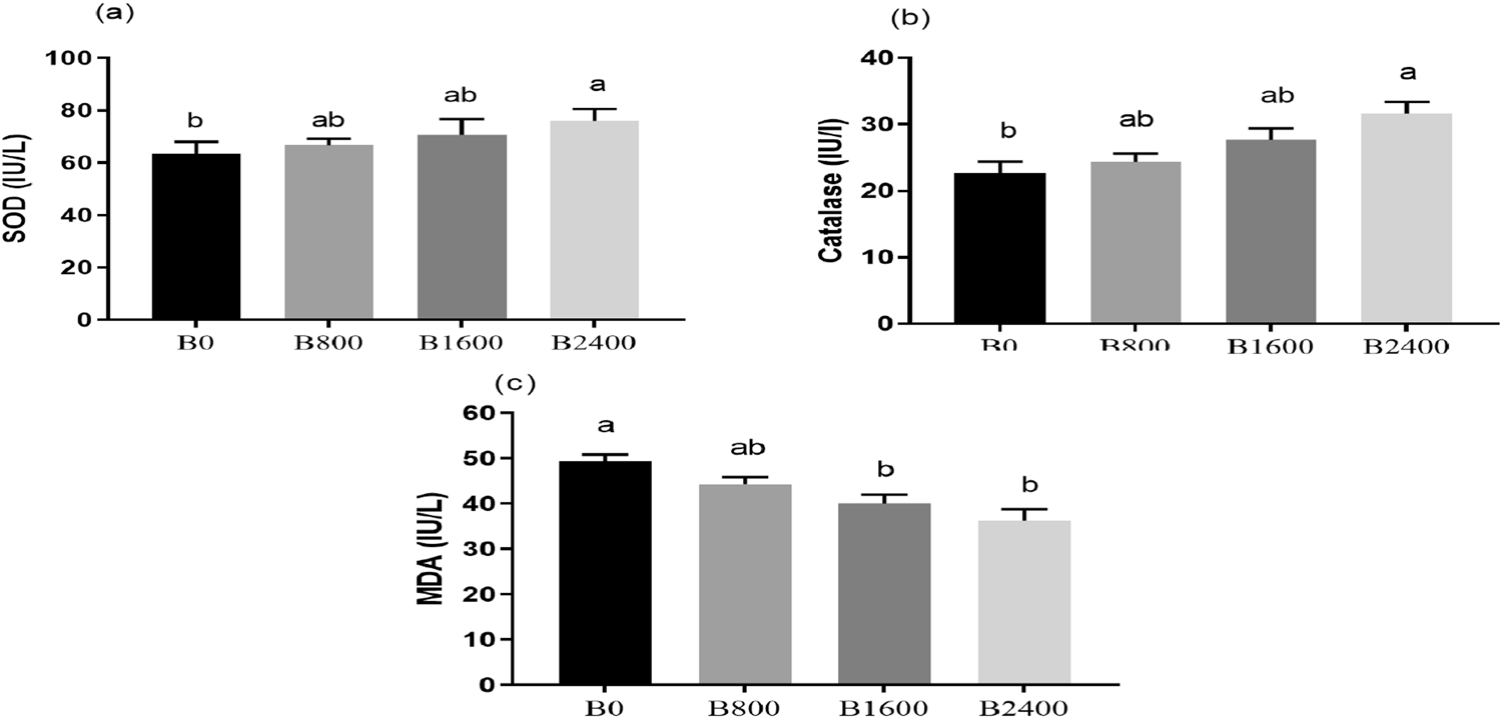
Oxidative parameters [SOD (**a**), catalase (**b**), and MDA (**c**)] of Nile tilapia fed experimental diets. The results were expressed as mean ± SE and the different letters indicate statistical significance at *P* < 0.05. SOD = Superoxide dismutase, MDA = Malonaldehyde.

**Fig. 7:**
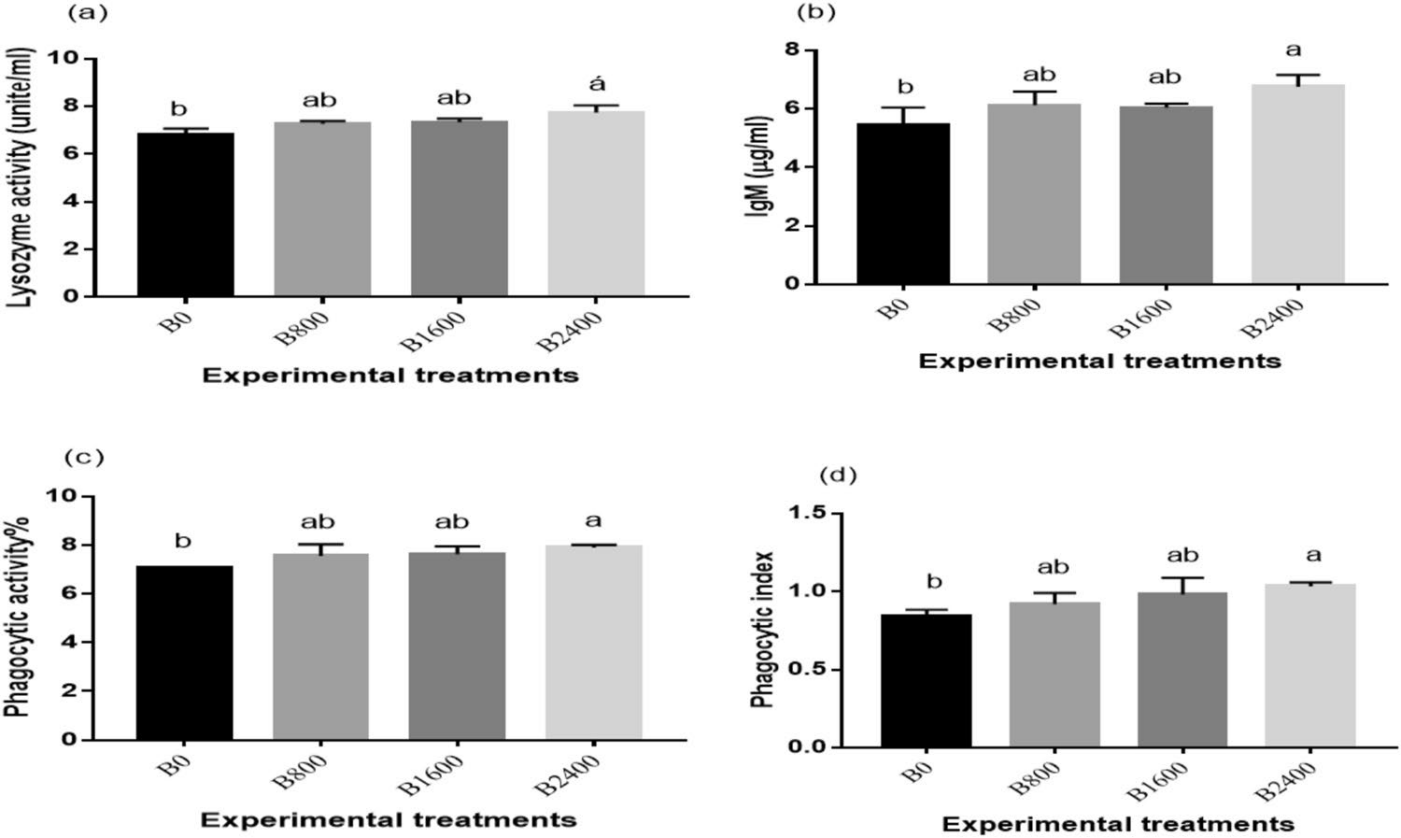
Immune response [lysozyme activity (**a**), Immunoglobulin M (IgM) (**b**), Phagocytic activity, (**c**) and phagocytic index (**d**)] of Nile tilapia fed different experimental diets. The results were expressed as mean ± SE and the different letters indicate statistical significance at *P* < 0.05.

The activities of the CAT and SOD enzymes were significantly boosted with increasing dietary betaine concentrations, with the maximum activities exhibited in the B2400 group (*P* = 0.022 & 0.047; Fig. 6a, b, respectively). Conversely, MDA concentration showed an opposite pattern with the peak values displayed in the control (B0) (*P* = 0.0002; Fig. 6c). Regarding the immune status, the lysozyme activity and IgM concentration, phagocytic index, and phagocytic activity were significantly elevated in the betaine treated groups in contrast to the control group, with the highest measure displayed by the B2400 group (*P* < 0.05; Fig. 7).

### qRT-PCR for some growth, antioxidant, and immune-related genes

**Fig. 8:**
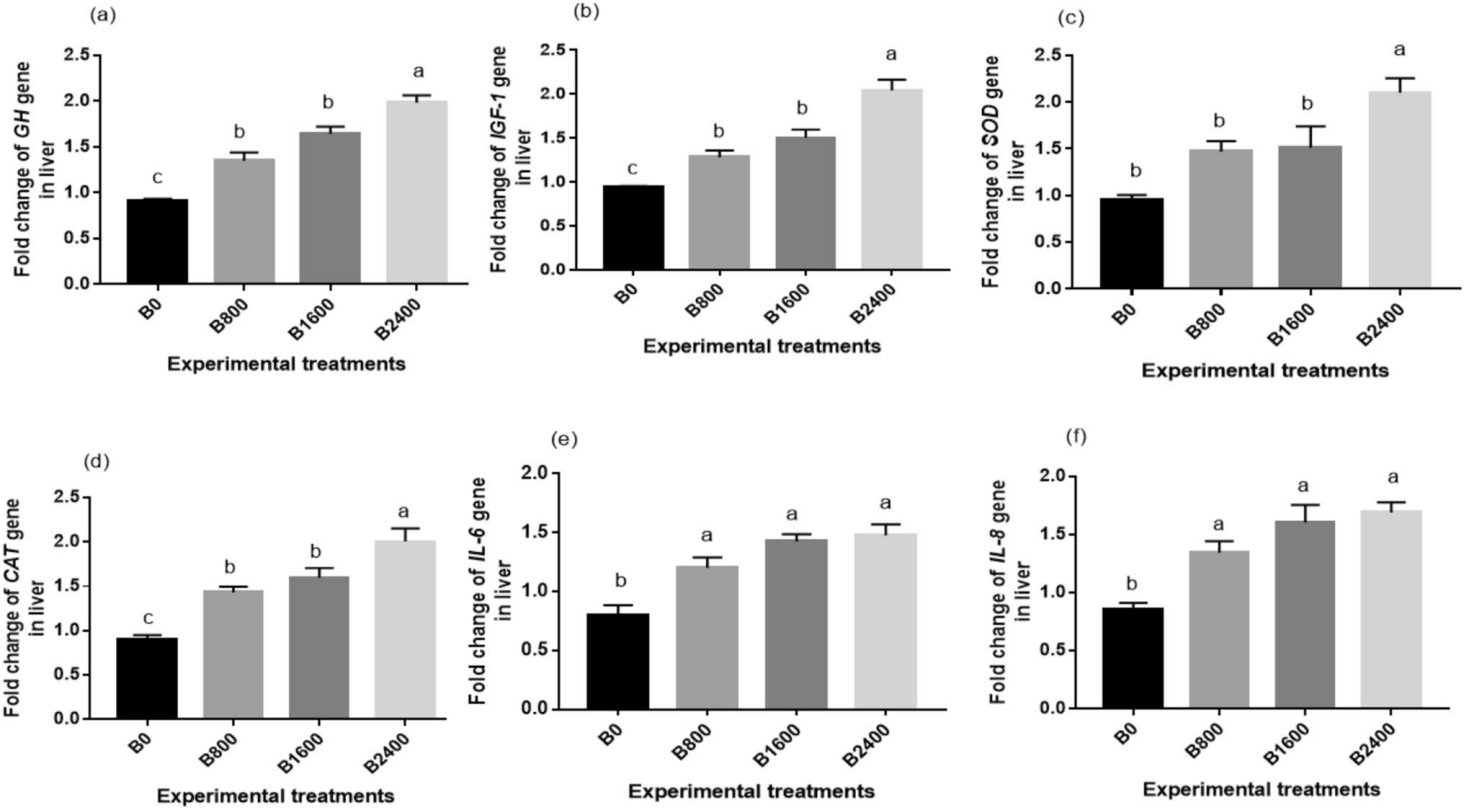
Transcriptomic response of liver selected genes [*Growth hormone (GH)* (**a**)*, Insulin growth factor 1 (IGF-1)* (**b**)*, Superoxide dismutase (SOD)* (**c**), *Catalase (CAT)* (**d**)*, Interleukin-6 (IL-6)* (**e**) and *Interleukin-8 (IL-8)* (**f**)] of fish fed different experimental diets. The results were expressed as means ± SE and the different letters indicate statistical significance at *P* < 0.05.

The results of the current work revealed that the expression levels of *IGF-1*, *GH*, *SOD*, *CAT*, *IL-6*, and *IL-8* in the liver tissues of *O. niloticus* were substantially affected by liquid betaine dietary inclusion (*P* < 0.05), compared to the control group (Fig. 8). The upregulations of *GH*, *SOD*, *IGF-1*, and *CAT* genes were significantly amplified as the dietary betaine level increased, reaching the maximum value at 2.4 ml kg^−1^ diet (Fig. 8a–d). Similarly, betaine supplementation triggered substantial increases (*P* < 0.05) in the activity of *IL-*8, and *IL-6*, in contrast to the control (Fig. 8e–f)*.*

### Acute ammonia challenge

#### Cumulative survival rate

**Fig. 9:**
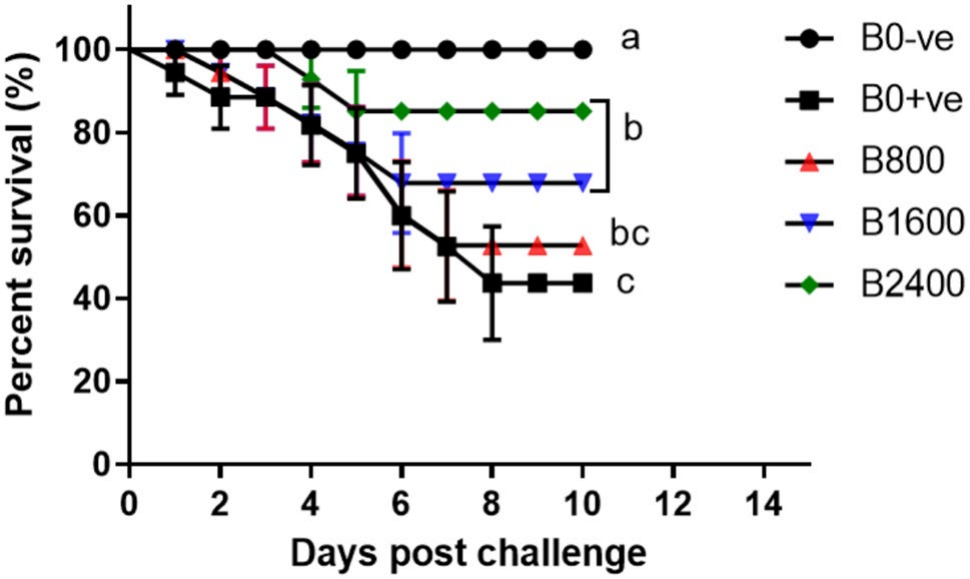
Log-rank (Mantel Cox) illustration of cumulative survival rate (%) of Nile tilapia fingerlings challenged with ammonia toxicity (ammonium chloride 0.07 g/litter for 72 h). Each line represents the means ± SE results of two parallel tanks holding 10 fish/tank. *P* = 0.025 as determined by Log-rank (Mantel Cox) test and *P* = 0.044 as determined by Gehan-Breslow-Wilcoxon test. Groups that do not share letters are significantly different at *P* < 0.05.

The cumulative survival rates of Nile tilapia fingerlings following 72 h of ammonia exposure were drastically altered by liquid betaine dietary supplementation (*P* < 0.05; Fig. 9). Throughout the 10 days observation period, the highest and lowest survival rates were observed in the B0-ve and B0 + ve groups (100% and 40%), respectively. Interestingly, the survival rates of the betaine-treated tilapia were around 80%, 70%, and 60% in the groups of 2400, 1600, and 800 betaine-supplemented, respectively.

### Histomorphology observations

**Fig. 10:**
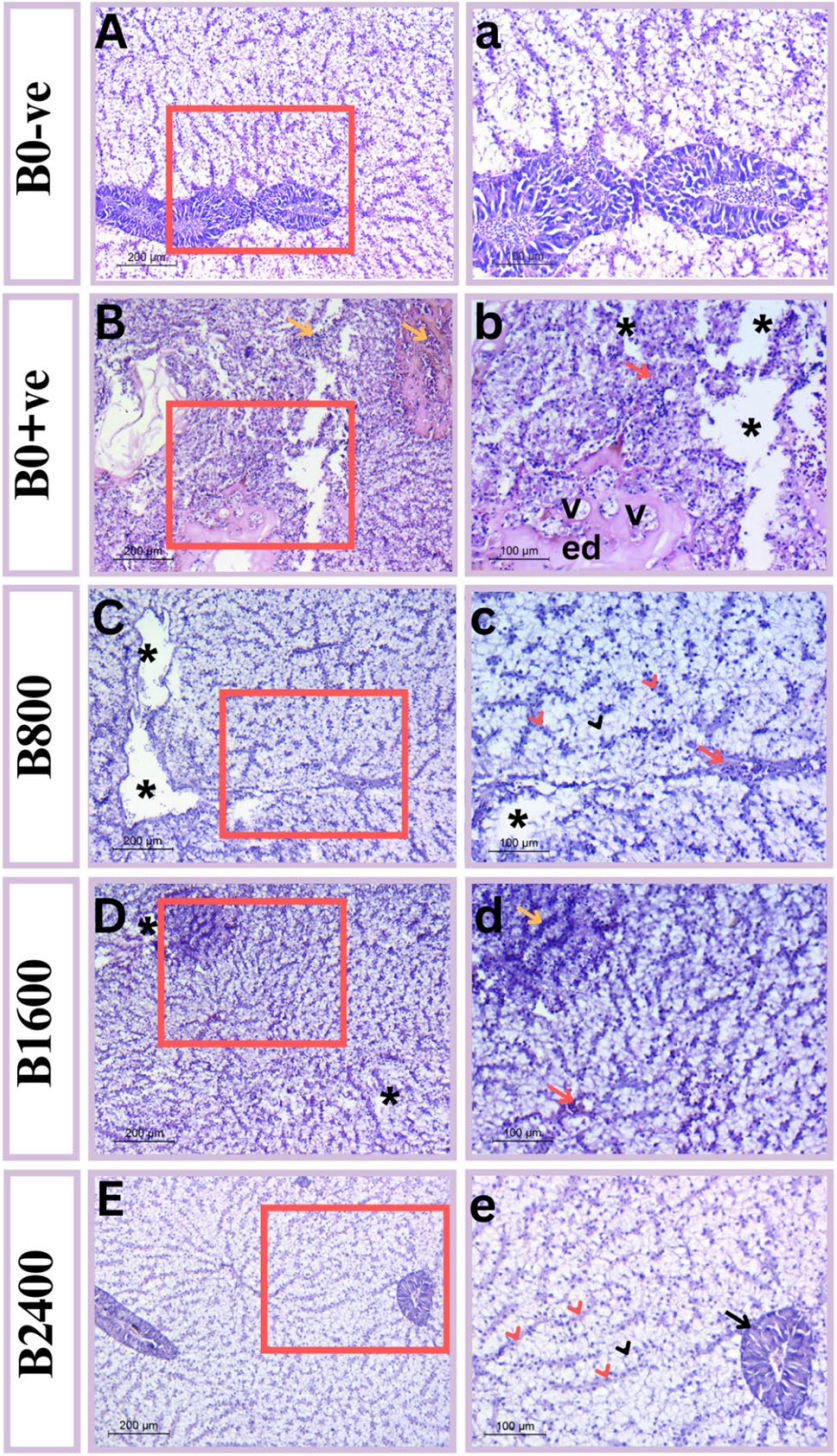
Photomicrograph of liver tissue showing (**A**,**a**) control group (B0-Ve) revealed normal histology of tissues; (**B**,**b**) positive control (B0 + Ve) liver of Nile tilapia showing hepatocytes degeneration (black asterisks), congested sinusoids (red arrow), vacuolated tissues (V), edema (ed) and leukocytes infiltrate (yellow arrow); (**C**,**c**) B800 treated liver of Nile tilapia showing altered hepatocytes (black head arrow), congested blood sinusoids (red head arrows), degeneration gaps (black asterisk) and dilated congested blood vessel (red arrow); (**D**,**d**) B1600 treated liver of Nile tilapia showing minimized number of vacuoles (black asterisks), leukocyte infiltration (yellow arrow) and small congested blood vessel (red arrow); (**E**,**e**) B2400 treated liver of Nile tilapia showing normal hepatocytes (black head arrow), normally blood sinusoids (red head arrow) and normal pancreatic acinus (black arrow). Tissues stained with H & E under magnification of 200 μ bar and 100 μ bar in which right panel represent higher magnification insets of left one.

**Fig. 11:**
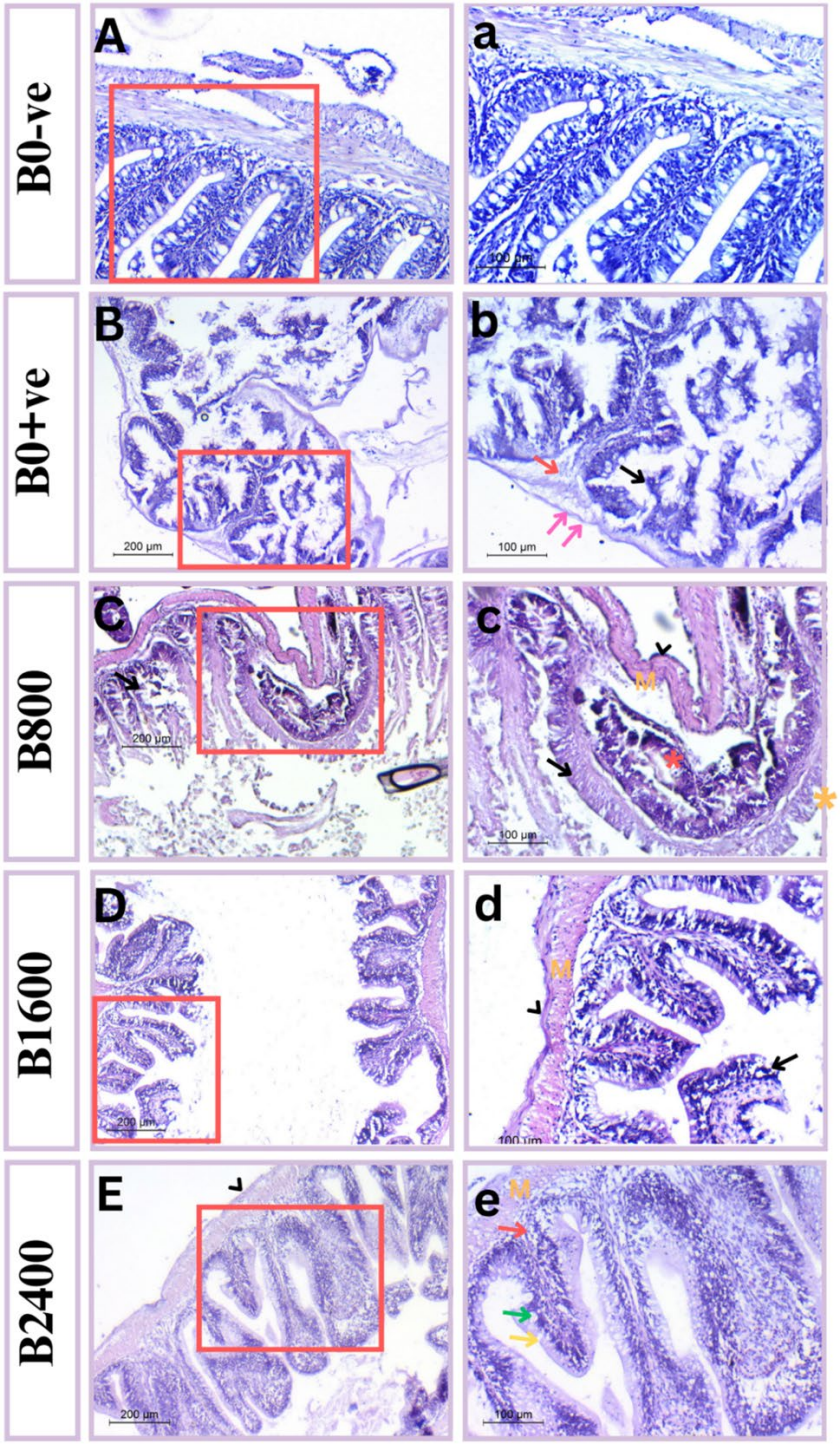
Photomicrograph of mid-intestine of fish showing (**A**,**a**) control group (B0-Ve) revealed normal histology of tissues; (**B**,**b**) positive control (B0 + Ve) showing villi degeneration (black arrow), submucosal edema ( red arrow), and muscularis and serosa disappearance (double pink arrow); (**C**,**c**) B800 treated fish showing thick serosa (black head arrow), developed muscularis (M), edema with eosinophilic granules (red asterisk) and developed villi (black arrow); (**D**,**d**) B1600 treated fish showing thick serosa (black head arrow), developed muscularis (M), leuckocyte infiltration (yellow asterisk) and sloughed villi with vacuoles (black arrow); (**E**,**e**) B2400 treated fish showing normal thin serosa (black head arrow), normally muscularis (M), submucosa (red arrow) and normal villi with goblet (yellow arrow) as well as lymphocytes (green arrow). Tissues stained with H & E under magnification of 200 μ bar and 100 μ bar in which right panel represent higher magnification insets of left one.

**Fig. 12:**
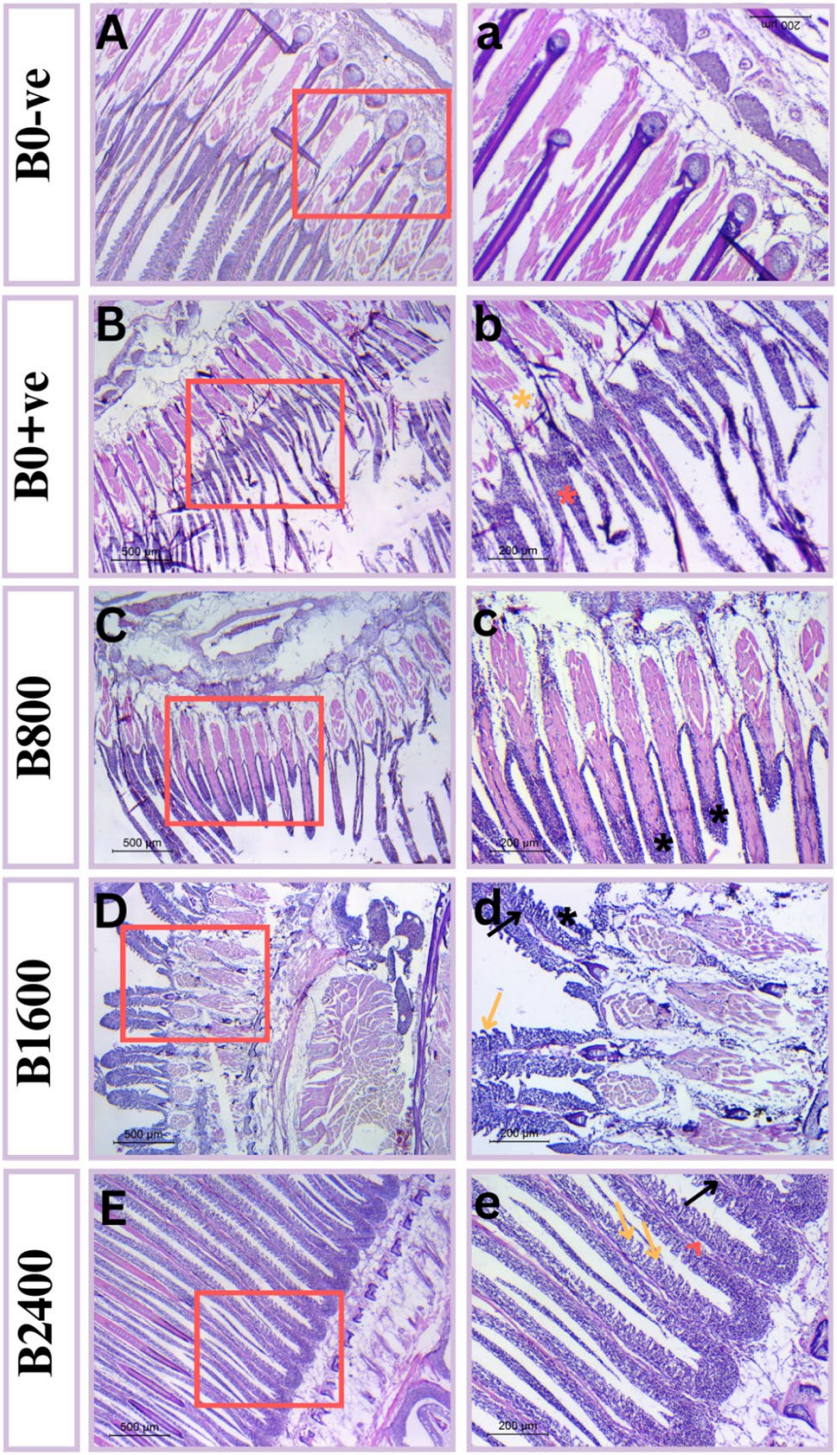
A photograph of Nile tilapia gills showing (**A,a**) control group(B0-Ve) revealed normal histology of tissues. (**B**,**b**) Gill of positive control fish, showing lamellar necrosis (red asterisk) and degeneration (yellow asterisk); (**C**,**c**) Gill of B800 treated fish showing development of lamellar filaments with fusion of adjacent secondary lamellae (black asterisk); (**D**,**d**) Gill of B1600 treated fish showing development of thick primary lamella (black arrow), clubbed tips filament (black asterisk) and separated thick secondary lamellae (yellow arrow); (**E**,**e**) Gill of B2400 treated fish showing normal developed primary lamella (black arrow) with appearance of vascular axis (red head arrow) and thin secondary lamellar filaments. Tissues stained with H & E under magnification of 500 μ bar and 200 μ bar in which right panel represent higher magnification insets of left one.

Our finding revealed that the effect of ammonia toxicity can be attributed to changes in cell shape, size and structure of fish liver, intestine, and gills. Furthermore, the histopathological data demonstrated that betaine exhibited dose-dependent protective effects against the acute ammonia toxicity observed in the three organs under histological investigation. (Figs. 10, 11, and 12).

For hepatic histopathological features, the control group (B0-ve) showed a normal mural structure with polygonal defined hepatocytes and a large spherical nucleus inside blood vessels identified as sinusoids. Exocrine pancreatic acini exhibited well-organized cytoarchitecture between hepatic tissues and a broad lumen (Fig. 10A, a). In contrast, the liver of positive- control fish (exposed to ammonia) reflected many signs of damage because of the acute toxicity of ammonia. These damage characteristics were manifested as irregularities in hepatocyte arrangement, sinusoids with blood congestion, and vacuolation dispersed across oedematous tissue. Additionally, leukocyte infiltration has been identified in the liver tissues, indicating the toxicity of ammonia (Fig. 10B, b). Hepatocyte anomalies with disintegrated cell borders were noticed in the B800 group. Numerous instances of tissue deterioration are still seen in the clogged blood sinusoids. Additionally, a large blood vessel that was congested was identified (Fig. 10C, c). A histological improvement was seen in the B1600 group with respect to the apparent decrease in deteriorated tissue. However, there are still clogged blood vessels and inflamed blood sinusoids visible (Fig. 10D, d). B2400-treated fish had the most typical look, which was described as spherically nucleated hepatocytes. Pancreatic acini have organized tissue with lumens. Blood sinusoids appeared darker due to their composition of erythrocytes (Fig. 10E, e).

For intestinal histopathological and morphometric characteristics, all betaine-fed fish groups displayed significant rises in the density, length, and branching of intestinal villi in a dose-dependent manner when opposed to the positive control (Table 6). The control group (B0-ve) showed normal cyoarchitecture of the intestine, as evidenced by normal thickness of numerous intestinal layers and lengthy villi of regular columnar epithelia (Fig. 11A, a). Positive control fish (exposure to ammonia) showed significant damage to all layers of intestinal tissue, demonstrated by an aberrant serosa layer, muscularis disappearance, villi deterioration, and submucosal edema (Fig. 11B, b). However, group B800-treated fish showed muscularis and villi development, but submucosal edema with few eosinophilic granular cells was also detected (Fig. 11C, c). Fish treated with B1600 exhibited thick serosa, normal muscle, and little leukocyte infiltration. Additionally, it was noticeable that the villi were shorter than typical, had fewer goblet cells, had patchy vacuolations, and were mildly sloughing (Fig. 11D, d). Other than that, the intestinal layers of the B2400-treated group showed no appreciable pathogenic alterations. Normal muscularis, submucosa, lamina propria, serosa, and mucosa with extended villi were seen in the gut. The villi epithelium was rich in lymphocytes and interrupted by many goblet cells (Fig. 11E, e).

**Table 6:** Intestinal histomorphometry of Nile tilapia (*O. niloticus*) fed with diets supplemented with various levels of Betaine for 60 days (n = 15) and challenged with acute ammonia toxicity.

	B0-ve	B0 + ve	B800	B1600	B2400	P-value
Villus height (μm)	65.367 ± 18.32^a^	15.43 ± 5.05^b^	17.56 ± 12.18^b^	32.43 ± 8.81^ab^	65.90 ± 18.40^a^	0.010
Villus width (tip) (μm)	18.07 ± 6.33^a^	6.53 ± 1.00^b^	9.3 ± 2.94 ^ab^	6.97 ± 1.35^b^	17.27 ± 6.128^a^	0.047
Villus width (base) (μm)	19.4 ± 6.02^a^	6.1 ± 3.23^b^	6.93 ± 0.19^b^	11.07 ± 2.51^ab^	19.33 ± 5.88^a^	0.020
Muscularis thickness (μm)	18.23 ± 6.01^a^	1.91 ± 0.64^b^	7.83 ± 1.32^ab^	10.57 ± 2.24^ab^	17.67 ± 3.86^a^	0.003

Means within the same row with different superscripts are significantly different at *P* < 0.05.

For gills tissue, the primary lamellar filaments and secondary lamellae, make up the gills showed typical thickness of the primary lamellar epithelium with one or two cell layers. The above-mentioned characteristics are demonstrated in the normal control group (B0-ve) (Fig. 12A, a). However, in the ammonia-exposed fish, the histological results revealed damages in the gill tissues (Fig. 12B, b) with an extensive and included necrosis. This damage resulted from lamellar degeneration, which led to complete lamellar fusions and the disappearance of vascularization. Only the extensions of the gill filaments and the beginning of tissue regeneration have significantly reduced these damage signs in B800, but clubbed tips were still visible (Fig. 12C, c). Furthermore, thick primary and secondary lamellar epithelial growth was seen in B1600 (Fig. 12D, d). By using a B2400 concentration, blood appeared in the vascular axis of primary filaments, as well as the primary and secondary lamellar development of normal thickness (Fig. 12E, e).

## Discussion

Enhancing the welfare of aquatic organisms via dietary control, including feed additives, is becoming a global trend that aims at attaining better growth metrics and efficient nutrient absorption^53,54^. Extensive research investigated the impacts of supplementation of synthetic betaine (betaine HCL) to fish basal diet. However, to our knowledge, limited studies have evaluated the influences of the natural form of liquid betaine on fish. In this study, we explored the effects of natural betaine (NATURA BETAINE) on growth performance, feed utilization, physiological and immuno-antioxidant responses under normal conditions as well as the alleviation effects to tissue damage because of acute ammonia challenge in Nile tilapia fingerlings.

Throughout the experiment, the supplementation of natural betaine resulted in a substantial boost in FW, BWG, and WG% in comparison with the control group (B0), with the best findings displayed by the highest concentration betaine group (B2400 group). The improvement in the growth performance of the B2400 group was accompanied by better feed utilization, in the form of the highest PER and SGR and lowest FCR. Additionally, based on polynomial regression analysis of FW, BWG, and FCR, the 2.4 ml kg^−1^ of dietary betaine level seemed to be optimal for *O. niloticus*. In agreement with the present findings, the supplementation of the synthetic betaine to the diet of GIFT strain of Nile tilapia resulted in a substantial increase in BWG, SGR^30^ as well as a rise in PER and reduction of FCR^55^. Additionally, dietary addition of synthetic betaine promoted the growth of rainbow trout^56^, giant freshwater prawn^57^, grass carp^58^, channel catfish^59^, mandarin fish^60^, gibel carp^61^, olive flounders^62^, *Labeo bata*^63^, and seabass^64^. The higher PER of Nile tilapia exhibited with a high dose of betaine supplementation may be attributed to the increased apparent crude protein digestibility^65^. The growth promoting effect of betaine may be related to the fact that betaine provides methyl groups to the fish, which are essential for the production of a number of substances, such as creatine and L-carnitine; therefore, it acts on the protein, energy and lipid metabolism^63,66^. Moreover, the fact that betaine enhanced the Nile tilapia growth supported by the induction of the digestive enzymes (amylase and lipase) activity and upregulation of growth-related genes (GH, IGF-1) as observed in the current results. In line with this result, synthetic betaine addition improved hepatopancreas lipase activity in prawn^57^ and gibel carp^29^, as well as intestinal amylase activity in juvenile tilapia^55^.

Variations in blood biomarkers can serve as valuable indicators for assessing the metabolic condition, immune function, and overall condition of fish^67,68^. The values of erythrocyte and leukocyte count were significantly improved with increasing dietary liquid betaine levels up to 2.4 ml kg^−1^ diet. This finding corresponds to^25^, who showed a similar effect using solid betaine in *Oreochromis niloticus*. Our data illustrated that feeding Nile tilapia fingerlings on betaine-based diets resulted in considerable upregulation in the levels of serum TP and globulins with the rise of the betaine dose. This outcome is consistent with that reported by^25^, who reported a significant improvement of TP following supplementation of synthetic betaine to Nile tilapia diet. Conversely, values of ALT, triglyceride, and cholesterol showed an opposite trend with the increase of betaine levels. Concurrent with these results, synthetic betaine addition significantly decreased serum cholesterol, triglyceride, and ALT in gibel carp^29^ and blunt snout bream^69^. Similarly, choline, a precursor of betaine, significantly reduced ALT activities of serum in hybrid grouper^70^. These results suggest that natural betaine could have an effective regulation on serum lipid metabolism as well as on maintaining the liver function. The betaine-linked regulation to lipid might be correlated with it methyl group donor ability^21^, influencing lipid metabolism through suppressing lipogenesis and enhancing lipolysis through modulation of different pathways such as protein kinase (AMPK) and mTOR which influence the levels of lipid metabolism-related enzymes like acetyl-CoA carboxylase (ACC), and fatty acid synthase (FAS)^71^. Therefore, future studies to explore the exact mechanism of betaine in altering serum lipids are recommended.

Water quality is considered one of the most critical factors that affect aquatic organisms health and productivity^72,73^. The water characteristics noted throughout the trial duration were found suitable for *O. niloticus* culture; however, they did not significantly differ among groups. In accordance with this result,^63^ observed that the inclusion of a synthetic form of betaine in feed did not influence the tank water quality. The improved water quality in the betaine-supplemented groups might be correlated to its physiological and metabolic impacts on Nile tilapia through enhancing feed utilization and reducing feed accumulation^25^ as well as lowering the nitrogenous waste such as ammonia and nitrite into water^74^. These effects, again, are associated with its ability to donate methyl groups that promote protein metabolism^21^.

Fish typically control reactive oxygen species (ROS) through a balanced interchange with antioxidant scavenging mechanisms. Nevertheless, disturbances produced by nutritional imbalances can upset this balance, triggering oxidative stress by disrupting the equilibrium between pro-oxidants and antioxidants^75^. To counteract the oxidative stress, fish have evolved antioxidant defense mechanisms such as antioxidant enzymes^54^. Of these antioxidant enzymes, CAT and SOD are the main elements involved in the antioxidant defense system through the elimination of the free radicals^76^. On the other hand, MDA is regarded as a critical indicator of the existence of oxidative stress and lipid peroxidation in fish^77^. In this study, natural betaine supplementation significantly improved the CAT and SOD enzyme activities in the serum of Nile tilapia, with the top figures exhibited in the highest concentration of betaine supplementation (B2400 treatment). On the contrary, MDA concentration showed a reverse pattern with the greatest values displayed by fish in the control group. Corresponding to the conclusions of this study, various findings pointed the enhancing effects of the synthetic betaine on the antioxidant enzymes of Nile tilapia^21^, zebrafish^78^, gibel carp^29^, mandarin fish^60^, blunt snout bream^69^, and prawn^57^. Additionally,^60,70^ reported that synthetic betaine and choline lowered MDA concentration in the liver of fish, respectively. The antioxidant activity of betaine may be related to the osmo- protection and methyl-providing properties^78^ as well as its role in the synthesis of reduced glutathione and thus safeguarding the cell from metabolites and ROS^79^. The reported increases in the antioxidant enzyme activities following liquid betaine supplementation under farming conditions might reflect priming or improved preparedness but not necessarily confirmed protection under oxidative challenge. Thus, future investigation of the regulatory effects of natural liquid betaine on antioxidant enzyme activities and their gene expression following different kinds of oxidative stress including ammonia stress are recommended.

The aquatic organisms’ innate immunity depends predominantly on several immune dynamics, including lysozymes, phagocytosis and IgM and therefore are regularly used as essential markers of fish immunity. In the existing study, the lysozyme, IgM, and phagocytic index and activity were substantially promoted in the betaine treated groups in comparison with the control group with the highest values displayed in the B2400 group. In accordance with the current findings,^62^ observed that serum lysozyme activity and leucocyte phagocytosis of tilapia were notably induced by solid betaine. Moreover, the immunostimulatory effect of betaine on fish has been previously reported in several studies, including grass carp^58^, ayu^80^, beluga^81^, and grouper^70^. The improvement of Nile tilapia immune status following betaine supplementation could be attributed to improved nutrient metabolism^62^. Additionally,^65^ attributed the immune-enhancement influence of betaine in Nile tilapia to the activation of melanomacrophage in the spleen.

The liver plays a crucial role in preserving the organism’s physiological equilibrium and defence against biological invasion^82^. Therefore, the assessment of growth and immuno-antioxidant-linked gene expression in the liver could be a valuable indicator of the fish health condition. This study revealed that the upregulations of *gh*, *igf*-1, *sod*, and *cat* genes were significantly enhanced in the liver of tilapia when the dietary betaine level increased, reaching the maximum value at 2.4 ml kg^−1^ diet. Concurrent with these results, a significant rise in the gene expression of *gh* and *igf*-1 following synthetic betaine supplementation was reported by^83^,^25^, respectively. These findings are aligned with the growth performance profile of fish in this study. Furthermore,^78^ showed a significant induction of the gene expression of *cat*, *sod* in zebrafish liver with the increase in synthetic betaine content in the diet. Increasing the expression levels of antioxidant-related genes by betaine could be attributed to its impact on the Wnt/β-catenin signalling pathway^78^. Our results showed that natural betaine supplementation resulted in a significant upregulation in the expression of *il-8,* and *il-6*, as opposed to the control group. Conversely,^70^ demonstrated that the expression of *il-6* was diminished considerably with the elevation of choline in the grouper’s diet. Overall, the gene expression results are consistent with our growth performance results and the trend of immune-antioxidant activity.

In aquaculture, ammonia is one of the detrimental compounds in water for fish survival and productivity, particularly when fish are grown in high density^84,85^. In the current study, cumulative mortality rates in response to ammonia toxicity were evaluated, and the survival rates of the betaine-treated groups were up-regulated in contrast to that of the control + ve treatment. Previous studies suggested that betaine could enhance the survival rates of fish against osmotic challenge^63^, viral diseases^62^, and vibriosis^80^. The higher survival rates exhibited by the natural betaine group could be due to the immunostimulant influence of betaine as observed in the current immune response results.

Liver histological examination can offer an indication about the nutritional and metabolic condition of fish^82^. Additionally, liver is considered the central component for the ammonia metabolism in fish^84^. The current study discovered that the best dose of natural betaine was 2.4 ml/Kg, which showed normal structure of liver tissues; nevertheless, notices of inflammation and hepatocyte deterioration were identified in the other groups. In line with the findings of the present trial, numerous experiments displayed the positive effects of solid betaine on liver histomorphology of carp^29^, and ayu^80^. In other study carried out by^70^, inclusion of choline in the diet of grouper attenuated the histological anomalies provoked by the rich-lipid diet. Moreover,^71^ attributed the protective role of betaine against liver irregularities to its involvement in the suppression of Sirt1/Pparɑ signaling pathway and, subsequently, weakened NF-kB associated inflammatory reaction in seabream.

Assessment of the morphology of the intestines is a guide of the digestive and absorptive ability of the aquatic organisms^67^. Our results exhibited a substantial boost in the length, density and branching of intestinal villi along with goblet cell count in the natural betaine supplemented groups in comparison with the positive control. Similarly, the findings of^25^;^65^ showed the same effect of synthetic betaine on tilapia gut. The enhancing influence of betaine on intestinal villi structures could be related to its capacity to act as an osmolyte, resulting in maintaining the integrity of villi and accordingly improving the digestibility and absorption of nutrients^21^. Additionally, betaine exhibited a dose—dependent improvement of intestinal structure against ammonia toxicity with the most typical look displayed in the high level (B2400) group. Concurrent with this result,^58^ detected a better morphology of the intestine in grass carp with betaine inclusion and thus improved enteritis morbidity. According to^86^, betaine enhanced the intestinal immunity of shrimp through promotion of the expression levels of intestinal *MYD88, PI3K, RELISH, IMD,* and *ROR* which play a key role in the resistance to pathogen- induced infection.

Gills are considered the target site of ammonia toxicity in fish water, which can evoke deterioration of respiratory activities^87^. Like the hepatic and intestinal histomorphology, consistent effects of betaine inclusion in diet were seen in gills, with the better lamellar structure exhibited in the B2400 group, while positive control group gills revealed the most impaired histological outlines manifested as necrosis, lamellar degeneration and fusion. Remarkably, these outcomes coincide with the pre-stress antioxidant action of the natural betaine and, consequently, this shields gills’ tissues from the deleterious induced consequences of free radicals and ROS^21,88^. Collectively, following ammonia exposure, there was clear reduction in the tissue alterations with higher survival rate in the betaine- supplemented groups, particularly at 2.4 mL kg^−^1. In a separate phase of this study (prior to ammonia exposure), improved basal antioxidant and immune status were reported. Although these findings may reflect a possible link, this study couldn’t provide a direct causal relationship between the reported improvement in the basal antioxidant status and post-ammonia stress outcomes. Thus, further studies are recommended to confirm whether improving the basal antioxidant status contribute to the reported post-ammonia resilience.

In general, despite the enhancement of Nile tilapia’s growth performance, hematological and biochemical profile, and the basal immune and antioxidant status in response to dietary supplementation with natural liquid betaine under normal conditions and its protective role to reduce the ammonia-associated tissue alterations in liver, gut and gills, several limitations should be acknowledged. First, the direct assessment of post-challenge biochemical response and the betaine’s ameliorating effects were not evaluated. Second, post-challenge antioxidant enzyme activities, oxidative stress biomarkers (e.g., MDA), and gene expression responses after ammonia exposure were not employed. Also, the ammonia challenge was conducted only for 72 h (acute model of stress), which may not fully reflect the chronic fluctuations in ammonia levels in commercial farming conditions.

## Conclusion

Overall, the results presented herein indicate that inclusion of 2.4 mL kg^−1^ natural liquid betaine in Nile tilapia fingerling diets has positive effects on the growth performance, feed utilization efficiency, hematological and biochemical indices, and immune-antioxidant status. Furthermore, it positively modulates the transcriptomic responses of several growth-, antioxidant- and immune-related genes under normal farming conditions. Additionally, the same dose of NATURA BETAINE improved the cumulative survival rate and mitigated the histopathological alterations in liver, intestine, and gills of tilapia following an acute ammonia challenge. The reported improvement of the basal antioxidant and innate immunity of Nile tilapia prior to ammonia challenge may have contributed to enhanced physiological resilience of Nile tilapia against acute ammonia stress. Collectively, these results suggest a guideline for setting effective dosages and delivery approaches for natural liquid betaine as a functional feed additive to ensure enhanced performance and stress preparedness in Nile tilapia aquaculture. Further studies evaluating post-challenge antioxidant enzyme activities, oxidative stress biomarkers (e.g., MDA), and gene expression responses after ammonia exposure to clarify the protective mechanisms of natural liquid betaine are recommended.

## Supplementary Information

Supplementary Information.

## Data Availability

The data included in this study can be accessed by contacting the corresponding author.
