# Supplementary figures and images for: Natural liquid betaine dietary supplementation improves growth performance, immuno-antioxidant responses, and stress resistance in Nile tilapia subjected to acute ammonia challenge
Source: Sci Rep. 2026 Apr 17;16:12706. doi: 10.1038/s41598-026-47150-0 (PMC13090391; doi:10.1038/s41598-026-47150-0)

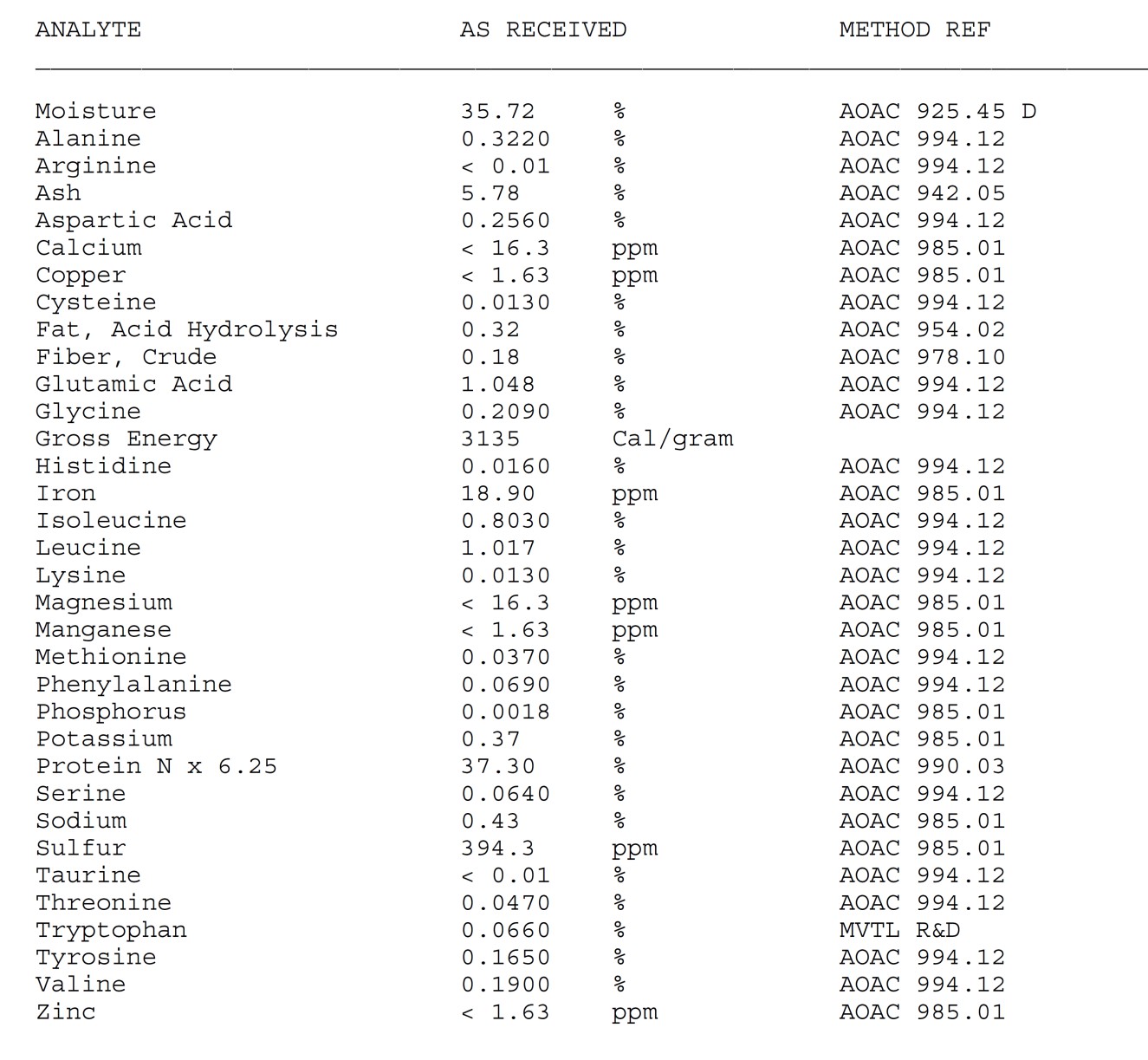

Supplement: Supplementary file 1 — Supplementary Information. [file 41598_2026_47150_MOESM1_ESM.docx]
